# Colour of medicines and children’s acceptability: What children think of the colour of oral dosage forms?

**DOI:** 10.3389/fddev.2026.1744120

**Published:** 2026-02-13

**Authors:** Elisa Alessandrini, Sveinbjorn Gizurarson, Jennifer Walsh, Roy Turner, Daniel Schaufelberger, Sonia Iurian, Hannah Batchelor, Sandra Klein, Begonya Nafria, Pamela Dicks, Segolene Gaillard, Axel R. Franz, Jasmin Albano, Aleksander Wisniewski, Charlotte Vermehren, Christina Gade, Jon Traerup Andersen, Catherine Tuleu

**Affiliations:** 1 UCL School of Pharmacy, University College London, London, United Kingdom; 2 Faculty of Pharmaceutical Sciences, University of Iceland, Reykjavik, Iceland; 3 Pharmacy Department, Kamuzu University of Health Sciences, Blantyre, Malawi; 4 Jenny Walsh Consulting Ltd., Nottingham, United Kingdom; 5 F. Hoffmann-La Roche Ltd., Basel, Switzerland; 6 School of Medicine, Neurology, Johns Hopkins University, All Children’s Hospital, St. Petersburg, FL, United States; 7 Department of Pharmaceutical Technology and Biopharmacy, Iuliu Hatieganu University of Medicine and Pharmacy, Cluj-Napoca, Romania; 8 Strathclyde Institute of Pharmacy and Biomedical Sciences, University of Strathclyde, Glasgow, United Kingdom; 9 Department of Pharmacy, University of Greifswald, Greifswald, Germany; 10 Institut de Recerca Sant Joan de Déu, Esplugues de Llobregat, Spain; 11 eYPAGnet, European Young Person’s Advisory Group Network, Barcelona, Spain; 12 NHS NRS Children’s Research Network, Royal Aberdeen Children’s Hospital, Scotland, United Kingdom; 13 CIC Inserm, Kids France, CHU-Lyon, Lyon, France; 14 Laboratoire de Biométrie et Biologie Évolutive, Université Lyon 1, CNRS UMR 5558, Villeurbanne, France; 15 Centre for Pediatric Clinical Studies, Universitätsklinikum Tübingen, Tübingen, Germany; 16 Department of Scientific Research, Children’s Memorial Health Institute, Warsaw, Poland; 17 Department of Drug Design and Pharmacology, Faculty of Health and Medical Sciences, University of Copenhagen, Copenhagen, Denmark; 18 The Capital Region’s Pharmacy (CV), Capital Region, Herlev, Denmark; 19 Department of Clinical Pharmacology, Copenhagen University Hospital - Bispebjerg and Frederiksberg, Copenhagen, Denmark; 20 Department of Clinical Medicine, Faculty of Health and Medical Sciences, University of Copenhagen, Copenhagen, Denmark

**Keywords:** acceptability, children, colour, oral medicines, preferences

## Abstract

**Background:**

Colour plays an important role in shaping consumer experiences, and in the context of medicines, it may influence patients’ expectations, behaviours, and treatment adherence. There is limited research on how children respond to medicine colour, despite their increased sensitivity to sensory cues. A previous literature review indicated that colour may affect medicine acceptability in children but highlighted the need for further research. This cross-sectional study aimed to fill that gap by directly collecting children’s opinions on the colour of oral medicines through an online survey, targeting a diverse paediatric population across age, health status, and countries in Europe.

**Methods:**

The survey, developed by UCL School of Pharmacy UCL REC (ID 26765/001) and reviewed by experts of the European Young Persons Advisory Group Network (eYPAGnet), was translated into five languages and distributed *via* QR codes and anonymous links. Response collection occurred between September 2024 and April 2025. Participants included children aged 3 to 18, with parental assistance when needed and parental consent. Responses were analysed using the software R Studio.

**Results:**

Out of 669 people who accessed the survey, 382 completed it. For liquids, pink (23%), colourless (16.5%), and blue (15.2%) were most preferred. Reasons included appealing look and taste associations (e.g., strawberry for pink). For solids, white (29.1%), pink (18%), and blue (12.5%) led, with neutral appearance and berry-related flavours cited. Statistically significant gender and age differences in preferences emerged, while variations by health, or country were not always significant. Ratings for previously used medicines highlighted preference for purple, pink, and blue, while white and colourless received moderate scores. A statistically significant link between taste and colour emerged (ρ = 0.42, *p-*value 2.2 × 10^−16^), suggesting that the colour of a medicine may influence children’s anticipation of its taste and shape their overall expectations of the treatment experience.

**Conclusion:**

This study offers valuable insight into children’s preferences for medicine colour. Pink and blue were associated with sweet/flavourful tastes, while neutral colours were perceived as tasteless, particularly by participants aged 12–18 years. These findings can inform paediatric formulation strategies, particularly the selection and use of colouring agents, in alignment with current regulatory requirements when their inclusion is considered necessary.

## 1 Introduction

The ability and willingness to take a medicine as prescribed, in short acceptability (European Medicines Agency EMA, 2012), is defined by several factors, some related to the user and others to the product itself. These attributes are often intertwined, and this makes the measurement of acceptability complex.

The appearance of a medicinal product such as its dimensions, shape, and colour, is among the first attributes observed by patients and may play a critical role in shaping their acceptance and willingness to adhere to treatment. In particular, the colour of medicines may play a critical role in shaping patient perceptions and preferences, especially during the initial administration, which can be particularly influential for children.

In addition, studies in adults have shown that colour can influence emotions, placebo effects, medication adherence, and even perceived therapeutic outcomes (Spence, 2021; de Craen et al., 1996; Tao et al., 2018; Srivastava and More, 2010; Mayor, 2013; Kesselheim et al., 2013; Amawi and Murdoch, 2022). The psychological impact of colour in adults is well documented, for instance, colours like red or orange are associated with energy and vitality, while blue or green may be more appropriate for tablets to reduce anxiety or promote relaxation (Spence, 2021; de Craen et al., 1996; Tao et al., 2018; Colorcon. Nutritional and Dietary Supplements, 2023). Findings also indicate that colour preferences vary by demographics such as age, gender, and region (Colorcon Nutra, 2023).

Little research has focused on children, despite indications of children being more sensitive than adults to sensory characteristics such as colour (Alessandrini et al., 2023). Children often have strong colour preferences and are naturally drawn to bright colours, particularly the youngest (Zentner, 2001; Boyatzis and Varghese, 1994; Pires and Agante, 2011). Therefore, in paediatric medicines, colour can be strategically used to reduce resistance to taking medicines. Nonetheless, it is essential to balance this appeal while avoiding an overly confectionery-like appearance to prevent unintended associations (Colorcon Nutra, 2023).

Beyond enhancing the aesthetical appeal of a product, colouring agents serve several practical functions. They can provide opacity to light sensitive active ingredients (Bhakti and Office of Cosmetics and Colors Center for Food Safety and Ap plied Nutrition Food and Drug Administration, 2023), or match the visual appearance of a formulation with its intended flavour (Alessandrini et al., 2023). Moreover, medicines are coloured for brand recognition and help patients identify and trust a particular manufacturer or brand, also adding a specific colour from a wide Pantone range can support identifying counterfeit drugs and makes counterfeiting more difficult. Last, colours can be used to facilitate identification of different strengths or formulations of the same or different products, to reduce the risks of medication errors (Alessandrini et al., 2023).

The literature does not clearly address whether colour plays different roles across dosage forms (e.g., liquid *versus* solid). We hypothesize that, similar to food and beverages where colour not only enhances visual appeal but also primes expectations of texture, flavour, and even temperature (Betina et al., 2016), colour in medicines may serve analogous functions. In liquid formulations, colour is likely to influence taste expectations and acceptance more strongly, whereas in solid dosage forms, its primary role may be in product recognition and reassurance rather than taste.

Colouring agents are widely used in over-the-counter products and dietary supplements, where they are primarily employed for marketing and consumer perceptions rather than because of any specific scientific rationale, helping products stand out in a competitive marketing (Colorcon Nutra, 2023).

In contrast, the use of colour additives in prescription medicines is strictly regulated, and rules vary across countries. For this reason, companies tend not to add colours to prescription medicines for children, unless necessary.

For instance, in Japan the colour options are typically much more limited than other countries because of regulations on dyes (Miller, 2015). In Europe, the use of colouring agents is regulated by the Directive 2009/35/EC (European Parliament, 2009) and the use of dyes in paediatric products is strictly regulated (European Medicines Agency EMA, 2012) and must be justified in terms of safety and potential benefits in the proposed patient population. In the United States, colour additives are subject to the FDA approval before they may be used in drugs for both adults and children (Code of Federal Regulations, 1977; Barrows et al., 2017) and likewise, this applies to other countries (Saito et al., 2022). Recently, a draft guidance from the FDA (Food and Drug Administration FDA, 2025) has outlined a more flexible approach that, under certain circumstances, allows manufacturers to easily replace colour additives in approved or marketed drug products.

The use of colours in paediatric medicines is further constrained by concerns, in certain countries, that certain dyes may cause adverse effects, such as allergic reactions or behavioural changes in children (Šuleková et al., 2017; First Steps Nutrition Trust). Although this paper does not aim to discuss the safety of colourants, it may contribute to the search for safer or more acceptable alternatives.

Moreover, the paediatric pharmaceutical market is more difficult to penetrate due to age-related research constraints and is also smaller compared to the adult market, thus limiting its research interest (Alessandrini et al., 2023).

A previous systematic literature review highlighted the scarcity of data on children’s views regarding colour of medicines, reinforcing the need for purposefully designed studies in the target population to fill these gaps (Alessandrini et al., 2023).

Therefore, we aimed to investigate children’s attitude and perceptions of the colour of oral medicines in a large and heterogeneous paediatric population across different age groups and countries in Europe to gain further insight into children’s views on the colour of oral dosage forms. Harnessing children’s views on their preferences could expand our knowledge on this aesthetic attribute, facilitating formulators’ choices and enabling informed decision-making regarding the need for or use for colours in paediatric products, in alignment with current regulatory requirements when their inclusion is considered necessary.

## 2 Materials and methods

This was a cross-sectional study conducted *via* an online survey. The questionnaire was developed in English by the UCL School of Pharmacy and reviewed by members of the WP4 Formulation Expert Group of concet4children (c4c, https://conect4children.org/) and experts of the European Young Persons Advisory Group Network (eYPAGnet), who piloted the survey with a group of young participants to ensure the contents and layouts were child-friendly and easy to understand by the target population. The pilot study included cohorts of adolescents and young adults aged 14–22 years from Spain, France, Denmark, Germany, and Poland. The number of participants and their age varied slightly by country, ranging from 6 to 12 individuals per cohort. During the pilot phase, by working in focus groups facilitated by the experts, young people from the eYPAGnet reviewed and adapted the survey. Following updates and finalisation, the survey and participant-facing materials (e.g., information sheets and consent forms) were translated by eYPAGnet experts into five further languages: Spanish, French, Danish, German, and Polish. These were countries where active members of the eYPAGnet group supported with translation and distribution. However, participation was not restricted to these countries, respondents from across Europe could complete the survey. All materials were uploaded into Qualtrics survey software (Provo, UT). Distribution was managed *via* anonymous links and QR codes, with eYPAGnet groups in each country promoting recruitment through various channels such as schools, hospitals, and social media. Due to the diversity of these channels, it was anticipated that responses will also come from countries other than those where a translation and active distribution were available. The survey ran from September 2024 to April 2025.

### 2.1 Inclusion and exclusion criteria

The study targeted children and adolescents aged between 3 and 18 years, if the child was unable to write or read, parental support was required. Colour recognition is generally considered a cognitive milestone for preschool children (3–5 years). By around age 3, most children can correctly identify and name a variety of colours (Davidoff and Mitchell, 1993; Mindful Me Behaviour Support). For this reason, our survey included children from the age of 3. However, literature also suggests that independent survey participation is typically feasible from about age 7 (Bell, 2007).

Children and adolescents were eligible to participate regardless of their health status or previous medication experience; even those who had never taken oral medicines were invited to complete the survey. This approach ensured responses from a heterogeneous group with different experiences with medicines.

There were no country restrictions for participation, despite the survey being translated into a limited number of languages to enhance comprehension. As the survey link was shared with multiple international collaborators *via* social media platforms such as LinkedIn, responses from disparate countries were expected.

### 2.2 Survey structure

The survey was designed to be completed anonymously and consisted of different versions tailored to the respondent:

- Young people (12–18 years) completed it independently.
- Children aged 7–11 years completed the survey with the help from an adult.
- For children aged 3–6 years, adults completed certain parts of the survey on their behalf, except for key questions where the child’s input was recommended.

In all cases, prior adult consent was required due to national laws of some countries where the survey was distributed. The three versions were structurally and content-wise very similar (see Supplementary Material). All consisted of close-ended questions, including either limited-choice and multiple-choice questions, some scalar questions (5-point hedonic scale), and some open-ended questions. All three versions consisted of 21 items and an adaptive questioning (Eysenbach, 2004) was used whereby certain items were conditionally displayed based on responses to previous items to reduce number of questions. In terms of contents, the first part of the survey focused on demographic information such as child’s age, gender, country of residence, chronic illnesses, and problems with colour vision. The second part asked questions about child’s familiarity with oral dosage forms and about the last oral dosage form taken (e.g., capsule, tablet, orodispersible tablet, minitablets, granules/powders, effervescent tablets, orodispersible film, and liquid medicine), visual images of each dosage form and explanations for some dosage forms were provided. Finally, the last section focused on hypothetical solid (represented as a tablet) and liquid medicines (represented as a bottle of syrup) and asked to provide preferences regarding their colour, taste, and reasons for the choice. The colour options included in our survey were informed by previous studies (Alessandrini et al., 2023), and by an evaluation of the colours of oral medicines included in the WHO Model list of essential medicines for children (ninth list, 2023) (World Health Organization). However, our final list did not include all possible hues, as an extensive range was considered too complex for young children to select from. Open questions were used to capture the taste evoked and reasons for participants’ choices. Parents completing or assisting with the survey were asked to allow their child to complete the final section independently, if possible. If the child needed help, parents were required to indicate this.

Participants could stop the survey at any point if they wanted to. The approximate completion time was 15 min.

The study was reviewed and approved by the UCL Research and Ethics Committee (ID 26765/001).

### 2.3 Statistical analysis and sample size calculation

The sample size was calculated using the standard formula for large populations, assuming a 95% confidence level, a 5% margin of error, and a conservative proportion estimate of 0.5, resulting in a minimum of 385 participants. Assuming an expected response rate of approximately 30% for online surveys, we estimated that at least 1,280 individuals needed to be contacted to achieve the target sample size of 385 participants.

Inclusion of participants’ data in the analysis was based on a completion rate of at least 90% of the survey questions. Forced-response validation was applied for key questions, requiring participants to provide answers before proceeding, to minimize missing data.

Data analysis and plots were performed using R and R Studio version 2023.06.0. To analyse the relationship between categorical variables, Fisher’s Exact or Chi-squared tests with simulations (based on 10,000 replicates) were used. Where a *p*-value was statistically significant (*p* < 0.05), analysis of standardised residuals was performed to identify which categories were contributing to the significance. Standardised residuals greater than 2 or less than −2 were set to indicate significant differences between the groups. Spearman’s rank correlation coefficient was used to analyse the relationship between Likert scale variables. To compare ratings across multiple groups Kruskal–Wallis was used, and Dunn’s test with Bonferroni adjustment as a *post hoc* pairwise comparison test.

Finally, for open-ended questions (i.e., reasons for choosing a colour for a hypothetical solid and liquid medicine and taste associated), the text was translated in English, if required, and cleaned. Then, a thematic analysis was conducted where recurring themes were identified and manually coded to categorise responses. Findings were then visualised using plots.

## 3 Results

### 3.1 Demographics

Out of the 669 responses received, 382 were included in the analysis.

Majority of participants were young persons aged 12–18 years (59.3%), whereas the others were children aged 7–11 years (21.3%) and 3–6 years completing the survey with the help of their parents (19.4%). The median age of children was 13 years (IQR: 8–15), with a range of 3–18 years. Participants self-identified as 56.5% female, 40.8% male, and 6.5% other/prefer not to say. In terms of health status, 20.9% had a chronic condition, 72.5% were healthy, and 2.6% did not know. Only 3.2% of the participants had a colour vision issue, whereas the great majority (94.2%) had normal colour vision and 2.6% did not know.

Approximately 80% of children completed the key section on colour preferences independently, while only 76 parents (20%) assisted their child with this part. This suggests that the majority of responses likely reflect children’s own view on the colour of medicines, enhancing the reliability of these findings.

Given the variety of channels used for distributing the survey, responses were received from disparate countries in Europe and fewer of them were from non-European countries. Table 1 provides an overview of the number of responses received from each region across Europe and outside, and of other demographic data.

**TABLE 1:** Demographic information about participants.

Demographics	Category	*N* (%)
Age	3–6 7–11 12–18	74 (19.4) 81 (21.3) 227 (59.3)
Gender	Female Male Other	216 (56.5) 156 (40.8) 10 (2.6)
Health status	Healthy Chronic condition Don’t know	277 (72.5) 80 (20.9) 25 (6.5)
Colour vision issues	Yes No Don’t know	12 (3.1) 360 (94.2) 10 (2.6)
Region	Eastern Europe *[Romania, Serbia, Albania, Poland]* Northen Europe *[Denmark, Sweden, UK, Ireland]* Southern Europe *[Spain, Andorra, Portugal, Cyprus]* Western Europe *[Germany, Austria, Switzerland, France, Belgium]* Outside Europe *[Canada, Paraguay, Singapore, Uzbekistan]*	49 (12.8) *[14, 1, 1, 32]* 68 (17.8) *[49, 3, 15, 1]* 118 (30.9) *[114, 1, 2, 1]* 142 (37.2) *[49, 3, 1, 89, 1]* 5 (1.3) *[2, 1, 1, 1]*

### 3.2 Children’s attitudes for the colour of hypothetical oral dosage forms

**FIGURE 1:**
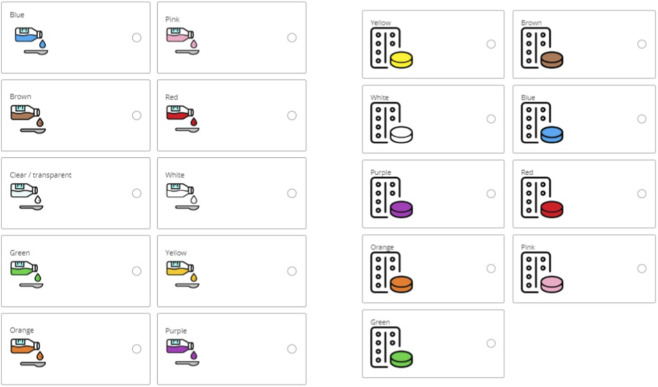
Images of coloured hypothetical liquid and solid medicines (tablets with blister) included in the survey.

Participants were asked identical questions regarding their preferences in terms of colour and taste for a hypothetical liquid and solid medicine which was represented as a tablet in the survey, Figure 1.

#### 3.2.1 Liquid medicines

For liquid medicines, the preferred colour was pink (23%), followed by colourless (16.5%), blue (15.2), purple (11.9%), orange (8.9%), red (8.7%), white (7.9%), green (3.5%), yellow (2.4%), and brown (1.9%).

When asked to indicate the reasons for choosing a specific colour, a multitude of motives were given and these differed depending on the colour. Overall, reasons in order of selection were favourite colour (132), neutral/natural (65), appealing colour (48), flavour association (44), familiarity (First Steps Nutrition Trust), symbolism (Code of Federal Regulations, 1977), emotional/psychological effect (Miller, 2015), no specific reason (Bhakti and Office of Cosmetics and Colors Center for Food Safety and Ap plied Nutrition Food and Drug Administration, 2023), avoidance of negative associations (Mayor, 2013).

**FIGURE 2:**
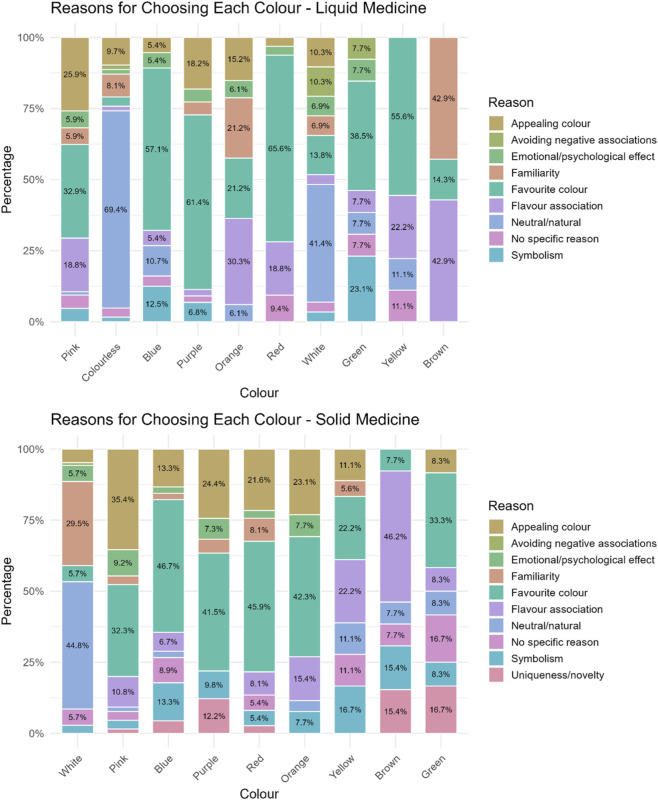
Reasons reported by participants for selecting a particular colour for their hypothetical liquid and solid medicine.

The main reason indicated by participants for choosing neutral colours, i.e., colourless and white, was that these colours were associated with a natural and/or neutral look (69.4% and 41.4% respectively), indicative of a non-toxic nor artificial product, “Because it is like water and so you do not think that it is a medicine” (colourless), “Other colours would look terribly chemical” (colourless), “It does not remind me of anything bad or dangerous to eat” (white), “It is neutral” (white). Choosing a colour because being their favourite was common, and most of participants selecting blue (57.1%), purple (61.4%), red (65.6%), and yellow (55.6%) selected them because of this, “I love it”, “It is my favourite colour”. Also, for pink and green, many selected these colours because of personal preference (32.9% and 38.5% respectively), however, others selected pink because it was an aesthetically pleasing colour for them (25.9%), “It is very aesthetically beautiful”, “It looks appetizing”, or because it reminded them of a positive flavour (18.8%), “Looks like candy”, “It reminds me of strawberries”. For green, aside from personal preferences, 23.1% selected it for symbolism, associative memory, “Nature and hope symbolized”. Interestingly, orange and brown, were mainly chosen because of flavour association (30.3% and 42.9% respectively), “Because I like the taste of oranges” (orange), “It can be chocolate” (brown), “Brown colour is very similar to honey” (brown), or familiarity with such colour (21.2% and 42.9% respectively), “Because it reminds me of the orange Dalsy [ibuprofen] which is very good” (orange), “It is the one in Doliprane [paracetamol] syrup for children which has a good taste” (brown), Figure 2.

Notably, some children (n = 6) reported deliberately avoiding colours that reminded them of medicines associated with past side effects. For example, an 18-year-old girl from Germany stated that: “The brain remembers the colour of the pill in conjunction with the side effects of the medication […] for a long time, even after stopping the medication, I could not drink orange juice or paint with yellow watercolours […]. So, if the pill is white, a connection between the colour of the medication and the side effects is not made as quickly”.

Reasons for choosing a colour varied depending on the age of the children, with younger participants (age groups 3–6 and 7–11 years) mainly selecting a colour because it was their favourite, appealing to them, or because of flavour association. Young people, instead, mainly favoured the neutral/natural look, although this was followed by their favourite/appealing colour.

**FIGURE 3:**
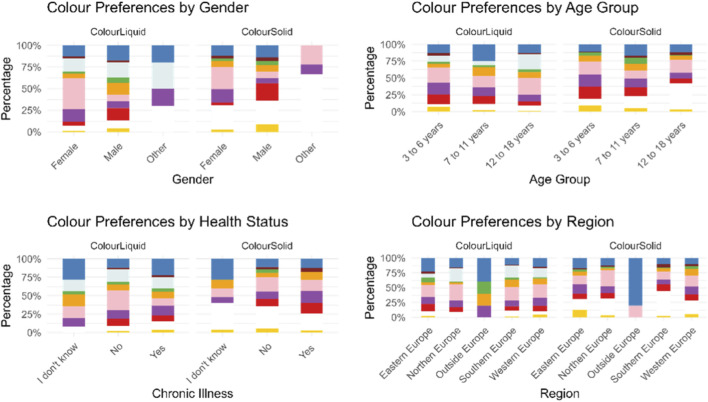
Participants’ colour preferences for a hypothetical liquid and solid medicine represented by gender, age group, health status, and geographical region.

Moreover, a statistically significant difference emerged across age-groups and colour preferences (*p*-value = 4 × 10^−4^), with analysis of residuals showing that colourless is strongly age-dependent being unpopular among younger children and strongly preferred by teenagers. Contrarily, red and yellow were more popular among younger children than adolescents. Blue was especially popular with 7–11 years old. Other colours had residuals close to 0, suggesting no strong differences among the age-groups, Figure 3 and Supplementary Table S1.

Results also indicated a statistically significant association between gender and colour preferences (*p*-value = 0.0001). Specifically, significant differences between males and females emerged for pink (*p*-value = 3.24 × 10^−10^), red (*p*-value = 0.002) and orange (*p*-value = 0.0152), Figure 3.

Instead, health status seemed to not affect the choice of participants, there was no statistically significant difference (p = 1) in the choices of participants with and without chronic illnesses, they all chose similar colours, Figure 3.

Across regions, European countries showed a similar preference for pink, while outside Europe (Canada, Paraguay, Singapore, and Uzbekistan) blue emerged to be the preferred colour, although the number of responses collected was very limited (n = 5). Slight regional variations were observed within Europe, but these differences were not statistically significant. In Eastern Europe (Romania, Serbia, Albania, and Poland), blue and green were chosen more frequently than in other countries, while colourless and orange were less common. In Northern Europe (Denmark, Sweden, United Kingdom, and Irland), colourless and pink were preferred, whereas orange and yellow were selected less frequently. In Southern Europe (Spain, Andorra, Portugal, and Cyprus), orange and colourless were more common than in other countries, while blue, brown, purple, and yellow were less common. Finally, in Western Europe (Germany, Austria, Switzerland, France, and Belgium), purple and yellow were chosen more frequently than anticipated, Figure 3.

**FIGURE 4:**
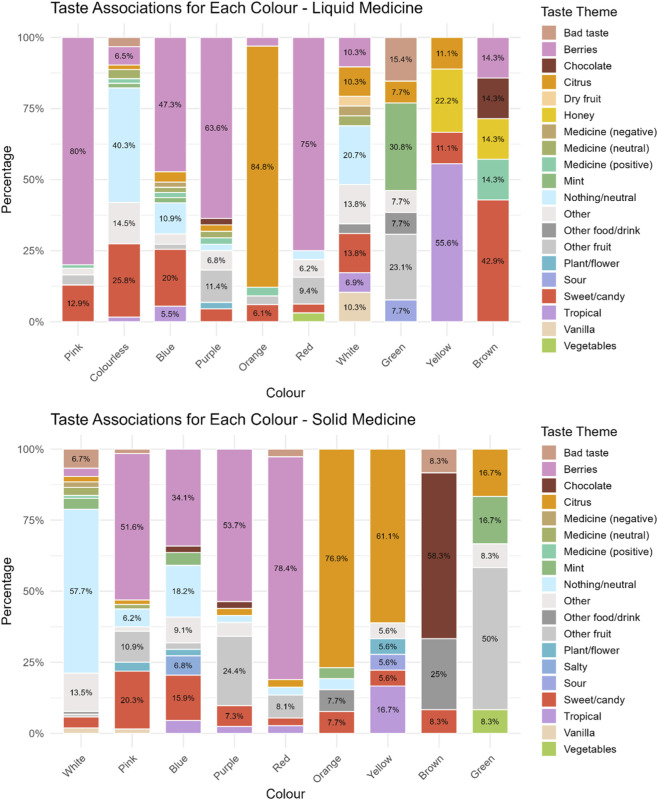
Taste associated to a particular colour for a hypothetical liquid and solid medicine.

Participants were also asked to indicate the taste they associated with the colour they had selected for the liquid medicine. Overall, berry flavours were the most frequently selected, with strawberry being the most popular. This was followed by sweet/candy flavours, neutral flavours, and citrus flavours, and then by the taste of various other fruits. Pink was largely associated with berry flavours (80%) (mainly strawberry 52/68 and raspberry 16/68), followed by a sweet, candy flavour (12.9%)). Similarly, purple (63.6%), red (75%) and blue (47.3%) were generally associated with a mix of berry flavours (blueberries, raspberries, strawberries, blackberries), with blue also linked to sweetness/candy (20%) or neutral flavours for 10.9% of participants who selected this colour. Colourless, instead, reminded most participants (40.3%) of a neutral taste (taste of water or nothing), followed by a sweet/candy flavour (25.8%). Orange was associated with an orange flavour by 84.8% of participants, whereas yellow was linked to a tropical flavour (banana 4/5, ananas 1/5) by 55.6% of participants selecting this colour, but to others it reminded of honey (22.2%). White, green, and brown inspired disparate taste/flavours. Interestingly, green was the only colour not associated with a sweet/candy flavour. Instead, about 30% of those selecting it associated this colour with mint. For the white colour, around 20% associated it with a neutral taste, others with sweetness, and 10.3% with a vanilla flavour, which was not evoked by any other colour. Brown was mainly associated with a sweet/candy flavour (42.9%), and in similar proportions with honey, a medicine with a positive taste, chocolate, or a berry flavour, Figure 4.

No significant differences emerged across age-groups in terms of taste associations, except for colourless liquid, for which younger children associated it with a sweet/candy flavour, 7–11 years mostly to a berry flavour, and 12–18 years to a neutral flavour.

#### 3.2.2 Solid medicines

For solid medicines, the preferred colour order was white (29.1%), followed by pink (18%), blue (12.5%), purple (11.4%), red (10%), orange (7.2%), yellow (5%), brown (3.6%), and green (3.3%).

Reasons for choosing a colour in order of selections were favourite colour (102), appealing colour (62), neutral/natural (54), familiarity (Jonauskaite et al., 2019), flavour association (Eysenbach, 2004), symbolism (First Steps Nutrition Trust), no specific reason (Saito et al., 2022), emotional/psychological effect (Code of Federal Regulations, 1977), uniqueness/novelty (Pires and Agante, 2011), avoidance of negative associations (European Medicines Agency EMA, 2012).

Reasons varied by age-groups. Participants aged 12–18 years mainly selected a colour because of its neutral/natural look, followed by appeal, and familiarity. In contrast, younger children (ages 3–6 and 7–11 years) mainly chose their favourite colour or one that was appealing.

The majority of participants who selected white explained that they chose it because of its natural look (44.8%), “It is neutral and non-alarming”, and familiarity of seeing solid medicines (tablets) in this colour (29.5%), “Colour of medicines”, “I am used to it”. Pink, blue, purple, red, and orange were selected mainly because it reminded participants of their favourite colour (32.3%, 46.7%, 41.5%, 45.9%, 43.3% respectively) or because it was an aesthetically pleasing colour for them (35.4%, 13.3%, 24.4%, 21.6%, 23.1% respectively), “Because it catches my attention and it is pretty”. Participants choosing yellow gave several reasons among which the most recurring were flavour association (22.2%), “It reminds me of lemon”, favourite colour (22.2%), and symbolism (16.7%), “It looks like the sun”. For brown colour the main reason was flavour association (46.2%), “It would have a chocolate flavour”, although some choose this for its uniqueness/novelty (15.4%), “I find it a curious colour”. Finally, main reasons for choosing green were favourite colour (33.3%), uniqueness/novelty (16.7%), “Because it is an interesting colour for a tablet”, although some did not really know (16.7%), Figure 2.

A statistically significant difference emerged across age-groups and colour preferences (*p*-value = 0.0001). An analysis of residuals showed that children aged 3 to 6 had a strong preference for red medicines, and slightly more than expected for purple and yellow. Children aged 7 to 11 showed a strong preference for green, and both groups were interested in white medicines. On the other hand, participants aged 12–18 years had a strong preference for white solid dosage forms, but a dislike for green, and less interest in purple and red medicines compared to the young participants, Figure 3 and Supplementary Table S1.

Results indicated a statistically significant association between gender and colour preferences (*p*-value = 0.0001). Specifically, significant differences between males and females emerged for pink (*p*-value = 4.8 × 10^−6^), red (*p*-value = 7.3 × 10^−7^), purple (*p*-value = 0.0063) and yellow (*p*-value = 0.0131), Figure 3.

Health status did not appear to affect participants’ colour choices. There was no statistically significant difference (*p*-value = 1) between those with and without chronic illnesses, Figure 3.

Significant regional differences were observed (*p*-value = 0.035). Specifically, outside Europe, the colour blue was chosen significantly more often than in other countries, although the sample size for outside Europe was much smaller compared to the others. In Northern Europe, pink was selected more frequently than in other countries. In Southern Europe, white was chosen significantly more often, while in Western Europe, orange was selected more frequently, and white was chosen less frequently than in other countries. Similarly, in Eastern Europe, fewer people selected white, whereas more people selected yellow, Figure 3.

With regard to taste associations, berry flavour was again the most frequently cited, followed by neutral taste, citrus, sweet/candy flavours, and other fruits. Majority of participants (57.7%) who selected white as a colour for solid medicines, stated that it should taste of nothing (“tasteless”, “neutral”), 13.5% selected other, which included “any flavour”, “just like everyone”, “indefinite”. Pink evoked a berry flavour in more than half of the participants who selected it (51.6%) (strawberry 23/33 and raspberry 10/33), for 20.3% of respondents pink was associated with sweetness, and for others to other fruits such as cherries, or fruit in general. Blue evoked mainly a berry flavour (mainly blueberry 13/15) (34.1%), followed by neutral/taste of nothing (18.2%), and a sweet/candy flavour by 15.9%. Purple and red were mainly associated with a berry flavour (53.7% and 78.4% respectively) (mainly strawberry flavour for most of those selecting red and a mix of berries for purple), followed by other fruit such as grape, plum, cherry, fig for purple, and watermelon and cherry for red colour. For majority of participants selecting orange and yellow, these colours evoked a citrus flavour (orange flavour for colour orange and lemon for yellow), and for 16.7% of participants yellow was associated with a tropical flavour, i.e., banana. Brown was instead associated with chocolate flavour (58.3%), for 25% it reminded of other food/drink such as Coca-Cola or a drink of “cinnamon, vanilla hot chocolate”. Finally, green mainly reminded respondents who selected this colour of fruits such as apple (3/6), kiwi (2/6), or watermelon (1/6), but also mint (16.7%), and citrus flavour (lime) (16.7%). No significant differences emerged across age-groups in terms of taste associations, Figure 4.

### 3.3 Colour perceptions of used oral dosage forms

Participants were asked to rate the colour, taste, and overall appreciation of the latest oral medicine taken, if they were currently taking more than one, they were asked the same questions for all oral dosage forms taken. Overall, participants took 236 prescription medicines, 190 over-the-counter (OTC) medicines and 15 did not know the distinction.

The extent to which colour and taste affected the overall appreciation of a medicine was evaluated. Overall, both colour and taste seemed to significantly impact the overall liking of a medicine. A moderate statistically significant positive association between colour liking and overall appreciation emerged (ρ = 0.53, *p*-value = 2.2 × 10^−16^). This indicates that colour liking is moderately, positively correlated to the overall liking of the medicines. Instead, taste emerged to be strongly, positively correlated with overall appreciation of a medicine (ρ = 0.7, *p*-value 2.2 × 10^−16^). Finally, a moderate positive correlation emerged between colour and taste (ρ = 0.42, *p*-value 2.2 × 10^−16^), indicating a statistically significant perceptual link between these two attributes.

**FIGURE 5:**
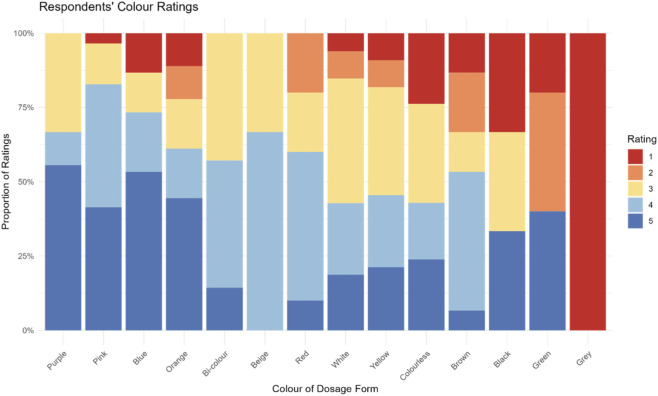
Participants’ ratings of colours of the most recently used oral dosage form. Ratings were based on a 5-point scale, where five indicated strong liking of the colour and 1 indicated strong dislike.

When assessing the average colour ratings for the last oral medicine participants’ took, it emerged that the colours with the highest rating (liked by participants) were purple, followed by pink, and blue. All three colours received an average rating equal or above four (indicative of liking). For all the other colours the mean rating was around 3, meaning that participants neither liked nor disliked the colour of the medicine they took. Only grey was disliked, however this was based on data from a single respondent, Table 2. A comprehensive picture of respondents’ colour rating is provided in Figure 5.

**TABLE 2:** Colours of most recently used oral dosage forms reported by participants, along with the adjusted mean ratings. Ratings were based on a five-point scale, where five indicated strong liking of the colour and 1 indicated strong dislike.

Colour	Mean rating	Count
Purple	4.22	9
Pink	4.17	29
Blue	4	15
Orange	3.72	18
Bi-colour	3.71	14
Beige	3.67	3
Red	3.5	10
White	3.4	262
Yellow	3.39	33
Colourless	3.19	31
Brown	3.13	15
Black	3	3
Green	3	5
Grey	1	1

An analysis by dosage forms showed that colour preference may vary depending on the dosage form. For instance, despite colour blue being not liked for liquid medicines and capsules, it was liked by the majority of those taking a tablet, orodispersible tablet/film, and minitablets (Supplementary Figure S1). However, it is difficult to draw solid conclusions because, for several colours, only one or two participants had taken medicines in that colour. The predominant colour of the dosage forms used by participants was white (61%). Ratings for white varied across dosage forms and were mixed, with some liking it (ratings ≥4), others disliking it (ratings ≤2), and others indifferent (rating = 3). Similar to the ratings for hypothetical medicines, children aged 12–18 years tended to give higher ratings (n = 168, mean rating 3.46, SD = 1) compared to the younger age groups: 7–11 years (n = 52, mean rating 3.35, SD = 1.12) and 3–6 years (n = 36, mean rating 3.14, SD = 1.38).

Finally, participants were asked whether they would be willing to take medicines without any colour (e.g., white or colourless), if necessary. These options were represented in the survey by images of a colourless liquid and white tablets. Of the respondents, 169 indicated they would be willing to take a colourless medicine, 120 said they would not, and 93 were unsure. An analysis by age-group showed that in the 3–6 years age group a similar proportion of respondents agreed and disagreed (41.9% vs. 40.5%). In the 7–11 years age group slightly more respondents were willing to take neutral colour medicines than those who were not (42% vs. 32.1%). For the 12–18 years old, more respondents were willing to take neutral colour medicines than those who were not (45.8% vs. 28.2%).

## 4 Discussion

Integrating children’s voices into the assessment of acceptability and preferences in the development of paediatric medicines is essential for creating products that truly meet their needs (Hauber et al., 2024), and research in this context has increased. However, there remains a lack of information specifically focused on the appearance of drug products, particularly regarding the colour of medicines as perceived by children.

This study offers a first comprehensive overview of European children’s attitudes and perceptions regarding the colour of oral medicines with few data also from other non-European countries, addressing a gap in the existing literature.

The experience of taking oral medicines is shaped by a combination of sensory attributes, including taste, texture, and visual appearance. Among these, colour, while rated as less important than palatability or swallowability (Alessandrini et al., 2021; Reidemeister et al., 2023; Ranmal et al., 2016), can still play a significant role in influencing acceptability, in paediatric populations.

Understanding how children perceive the colour of medicines is therefore essential for developing formulations that are both effective and acceptable, ultimately supporting better adherence.

Our findings confirm that while taste is the strongest determinant of product acceptability, colour also demonstrates a moderate positive correlation. Children who liked the colour of their medicine tended to report greater overall liking of the product. However, this correlation does not necessarily imply direct effect, and multisensory integration likely mediates the relationship.

Moreover, our results indicate that children may psychologically or sensorially associate certain colours with certain tastes, even before tasting the medicines, and this could influence their overall acceptability of the medicine. Evidence from the food industry shows that consumers often associate specific colours with particular tastes and flavours (Spence and Levitan, 2022) and our results suggest the same applies to medicines. For example, many children in this study expected a pink medicine to taste of strawberries or something sweet, while an orange medicine was associated with an orange flavour. Thus, colour appears to carry an emotional component, priming children’s senses for a certain taste. This may make medicines feel less intimidating and enhance children’s expectations of the treatment experience in some cases. However, it is essential to balance this appeal while avoiding an overly confectionery-like appearance to prevent unintended associations (Colorcon Nutra, 2023).

On the other hand, the colour of a medicine can also be associated with a negative experience, potentially preventing a child from taking future medicines of that colour. This suggests that negative experience with a medicine of a particular colour may strongly affect trust in other medicines of the same colour.

The choice of which colour to use depends on several factors. The type of dosage form may play a role, as some colours may be better accepted for certain dosage forms than others. For instance, white is more accepted for solid medicines (e.g., tablet) than for liquid medicines, whereas brown is more appreciated for liquid medicines than for capsules. However, in this study, attitudes for hypothetical solid and liquid medicines were similar. Pink, blue, and neutral colours (colourless/white) emerged as the most preferred for both dosage forms. Neutral colours, although often selected as favourite for hypothetical medicines, received average ratings when children evaluated medicines they had already taken. This likely reflects acceptance, with children finding the colour acceptable but without strong feelings either way.

Age was a key factor influencing colour choices. Younger children tended to prefer bright, playful colours such as red or yellow, consistent with previous research showing that children are naturally drawn to lighter and happier colours (Zentner, 2001; Boyatzis and Varghese, 1994). In contrast, adolescents favoured neutral tones such as white, often linking them to familiarity and perceived seriousness of the medicines. For example, a 13-year-old boy from Denmark wrote: “that’s the colour I’m used to”, and a 13-year-old boy from Spain explained that “for me it is the standard tablet colour and when I see another colour it makes me feel sceptical of that tablet”. This suggests that colour preferences evolve with psychological development and medicine-taking experience, and could inform an age-stratified product design in paediatric formulations.

Previous research on children’s colour preferences (Zentner, 2001) suggests that an important developmental transition in colour attractiveness occurs during the preschool and early elementary years, likely influenced by increased social contact and schooling. This may explain the shift in preference from red to blue observed around the age of 7 years and which was noticed in our study as well. The decline in preference for red may reflect its cultural ambivalence, as red is often associated not only with positive attributes but also with unpleasant concepts such as blood or danger (Zentner, 2001). Moreover, not all bright colours appear equally attractive in the context of medicines. In this study, yellow and green, were chosen as favourite by a smaller proportion of children compared with red or blue. The reasons for these differences are not entirely clear but may relate to flavour associations: for example, red was commonly linked to sweet or fruity tastes, whereas green was sometimes associated with vegetables such as broccoli, which are generally less appealing to children than fruity flavours. Moreover, green was the only colour not being associated with sweetness, which may explain why it was less selected than other colours. A large study conducted with 5,000 participants assessing taste association elicited by six coloured drinks, reported that the sweetest of the drink’s colours shown was red, closely followed by blue and then purple. By contrast, green, yellow, and orange were rarely chosen as colours associated with sweetness (Spence, 2019). This indicates that some colours more than others are associated with sweetness hence more appreciated by children also for their medicines.

The reasons for choosing a specific colour differed by age group. Children aged 3–11 years often chose colours because they were their favourites or appealing to them. Flavour association (e.g., strawberry for pink) was also an important reason for this group. A study exploring the links between colours and taste/flavours, reported that young children, similarly to the elderly, rely more on visual cues when trying to identify the taste of food and drink than adults (Spence and Levitan, 2022). Adolescents, however, placed more emphasis on neutrality, and familiarity with previous medicines. This shift indicates that while bright and appealing colours may encourage younger children to accept medicines, older children and adolescents may prefer subtler, more traditional colours that inspire trust and familiarity.

Gender also appeared to influence colour preferences, with pink and purple predominantly liked by females, while males showed preferences for red and orange in liquid medicines and red and yellow in solid medicines. Other colours were similarly preferred across genders. These differences may be linked to social and cultural differences, such as marketing and gendered colour associations (LoBue and DeLoache, 2011; Wong and Hines, 2015). A study evaluating colour preferences in a sample of children and adults reported similar results to what identified in our study: pink was mainly liked by females, red by males and blue by both. The authors speculated that these differences should account emotional associations but also gender stereotypes and status differences between men and women (Jonauskaite et al., 2019). Interestingly, the flavours associated with these colours were consistent across genders, with strawberry, and berry flavour more generally, the most frequently mentioned.

No significant differences emerged by health status, although children with chronic conditions represented only a small proportion of the sample.

Regional differences across Europe were limited. Orange for instance was more commonly selected in western and southern countries, while pink was more popular in northern countries. These trends may reflect exposure to widely used medicines in local markets, such as orange-coloured Dalsy in Spain, as reported by some participants: “Because it reminds me of the orange Dalsy which is very good” (girl, 15 years, Spain), or orange solid forms: “Because it is the colour of the vitamins I already have to take” (boy, 10 years, France). Blue was popular outside Europe, although responses from non-European regions were limited. Further research including larger samples from outside Europe would be valuable to better understand the extent of geographical variations in colour preferences and align with cultural expectations. Studies in the food context show that people of across different cultures sometimes associate different flavours to the same colour, for instance young Taiwanese consumers tested in one study expected a transparent blue drink to taste of mint, whereas young British consumers expected a raspberry-flavoured drink instead (Spence, 2019). Cultural differences in how ingredients are used in cuisine may influence taste and flavour expectations (Spence and Levitan, 2022) and this may happen as well in the pharmaceutical context and needs to be kept in mind. A good example in the pharmaceutical filed is the Tylenol (paracetamol/acetaminophen) rebranding in Japan, where, red signifies respiratory medicine, not pain relief. Tylenol changed its packaging to blue for adults and pink for children, avoiding confusion and aligning with local associations. This change followed local market research and significantly improved acceptance (Lynk, 2006).

Compared with the findings of a previous literature review (Alessandrini et al., 2023), this study contributes several new insights into children’s attitudes and perceptions of medicine colours, particularly regarding the effect of gender, age, health status, dosage form, and regional differences. Interestingly, some discrepancies emerged between findings from this study and those from the literature review. For example, while blue had previously been reported as a colour rarely chosen by children, it ranked among the top three favourites in our study, both for hypothetical preferences and for medicines already taken. Conversely, red, identified in the literature as the most favoured colour, consistently ranked in the middle, suggesting it is generally liked but not as strongly as other colours. This difference may reflect the limited amount of data available and different methodologies applied in the studies collected in the literature review. Furthermore, our results confirmed the well-established association between pink and strawberry flavour, reinforcing its relevance for paediatric formulations at least in the European market.

This information, when considered alongside country-specific regulations on colouring agents and an understanding of the potential risk-benefit ratio of including colour in paediatric formulations, may support formulators in selecting appropriate colour and flavour profiles for children’s medicines.

The types and usage of dyes in paediatric medicines are strictly regulated in many countries. As a result, for prescription medicines, formulators are often reluctant to include colouring agents, as their use must be clearly justified. In contrast, commercial products such as over-the-counter products and food supplements are often coloured for marketing purposes. However, the exact quantities of dyes used in such commercial products, are typically proprietary. This lack of transparency makes it challenging to assess dietary intake and evaluate exposure levels in children. This may be particularly concerning given the potential health-related issues associated with certain dyes, including genotoxicity, allergic and asthmatic reactions in sensitive individuals, headaches, and hyperactivity in children (Šuleková et al., 2017; First Steps Nutrition Trust).

A study assessing certified food dye use in over-the-counter medicines and supplements for children and pregnant women found that intake of Red No. 40 (Allura Red, disodium 6-hydroxy-5-((2-methoxy-5-methyl-4-sulfophenyl)azo)-2-naphthalenesulfonate) exceeded the U.S. FDA’s acceptable daily intake (ADI) by two to three times in some children’s pain relievers and cough/cold/allergy syrups. These findings raise questions about the extent to which dyes may influence behaviour in children and highlight the need for more transparent labelling and exposure assessment (Lehmkuhler et al., 2020).

This study had some limitations. First, as it was conducted online, it was not possible to verify the reliability of participants’ responses, although in some countries like Spain the questionnaire was also disseminated in schools, providing some reliability to the data. This is a common limitation of studies using this methodology. Nonetheless, using an online survey provided a faster and relatively simple way to reach a large, heterogeneous population, compared to a face-to-face study of similar scale, which would have been costly and impractical.

Furthermore, participants were asked to select the colour of a hypothetical liquid and solid medicine based on images, which may not fully reflect their reactions to an actual medicine of the same colour in real life. To address this, we also asked them to rate the colour and their liking of the last oral medicine they had taken. This allowed us to compare hypothetical preferences with real-world experiences, and the order of preferences was similar across both.

Online surveys often struggle to achieve the large sample sizes initially anticipated. In this case, distribution relied on eYPAGnet group members in each country, and the number of responses varied depending on the reach of its members. Nonetheless, the final sample size was sufficient to identify trends and generate valuable insights into European children’s preferences.

A small number of responses were also collected from outside Europe, although this was not the primary aim of the study. Expanding future research to other geographical locations, such as Asia, would be valuable to examine whether colour preferences, reasons for choice, and flavour associations align with or differ from the findings in this study.

## 5 Conclusion

This is the first patient-centric and comprehensive study to provide valuable insights into children’s views on the colours of oral medicines across multiple European countries, with some additional data from non-European countries, thereby addressing a gap in the existing literature. The findings indicate that colour is moderately correlated with medicine acceptability and, in children, it is linked to taste perception. The colour of a medicine appears to prime children’s sensory expectations, influencing their anticipation of taste and making the medicine more or less intimidating, depending on their previous experience. Preferences vary with age, brighter hues are favoured in younger children, whereas adolescents prefer neutral/white. Gender differences and some regional variations were also observed, highlighting additional factors worth consideration. Broader, cross-cultural studies are needed to verify generalizability to better inform the design of paediatric medicines in a global market.

## Data Availability

The raw data supporting the conclusions of this article will be made available by the authors, without undue reservation.
