# Correction: Colour of medicines and children’s acceptability: What children think of the colour of oral dosage forms?

**DOI:** 10.3389/fddev.2026.1818974

**Published:** 2026-03-18

**Authors:** Frontiers Production Office

**Affiliations:** Frontiers Media SA, Lausanne, Switzerland

**Keywords:** acceptability, children, colour, oral medicines, preferences

Reviewer Garba Mohammed Khalid was erroneously listed as being affiliated with Queen’s University Belfast, United Kingdom. The correct affiliation is Kingston University London, United Kingdom.

The original article has been updated.

