# Supplementary material for: Colour of medicines and children’s acceptability: What children think of the colour of oral dosage forms?
Source: Front Drug Deliv. 2026 Feb 13;6:1744120. doi: 10.3389/fddev.2026.1744120 (PMC12946078; doi:10.3389/fddev.2026.1744120)
Supplement: Supplementary file 1 [file Supplementaryfile1.docx]

Supplementary Material

# Supplementary Data

Colour medicines survey

Start of Block: Block 1 - Who's completing the survey

Q2 Who is completing this questionnaire?

- I am the adult of a child aged **3 to 6 years** old (4)
- I am the adult of a child aged **7 to 11 years** old and I am completing this survey with my child (2)
- I am **12 to 18** years old (3)

End of Block: Block 1 - Who's completing the survey

Start of Block: Block 2 - PIS and Consent adult

Display this question:

If Who is completing this questionnaire? = I am the adult of a child aged <strong>7 to 11 years</strong> old and I am completing this survey with my child

Or Who is completing this questionnaire? = I am the adult of a child aged <strong>3 to 6 years</strong> old

Q24 Please read the Information Sheet to learn more about this survey. We have designed a document with information about this study for your child too.  Depending on their age, please click on the links below: For children LESS than 6 years  For children 6 to 11 years **After reading these documents, please indicate if you and your child agree to take the survey.**

- I AGREE for myself and my child to take part in this survey (1)

End of Block: Block 2 - PIS and Consent adult

Start of Block: Block 3 - PIS and consent young person

Display this question:

If Who is completing this questionnaire? = I am <strong>12 to 18</strong> years old

Q23 Before starting the survey, please learn more about this survey.  Depending on your age, click on one of the links below: - ADULT CAREGIVER - 16 TO 18 YEARS - 12 TTO 15 YEARS **After you have read the documents, please indicate: If you are happy for your child to take the survey (adult)**

- I am the adult and I AGREE for my child to take part in this survey (3)

Display this question:

If Who is completing this questionnaire? = I am <strong>12 to 18</strong> years old

Q86 **If you are happy to take the survey (young person)**

- I am HAPPY to take the survey (1)

End of Block: Block 3 - PIS and consent young person

Start of Block: Block 4 -Survey description for adults

Display this question:

If Who is completing this questionnaire? = I am the adult of a child aged <strong>3 to 6 years</strong> old

Q39 The survey is divided in **two parts**: PART 1: to be **completed** by the **adult** PART 2: to be **completed** by the **child with** the **help of the adult**

Display this question:

If Who is completing this questionnaire? = I am the adult of a child aged <strong>7 to 11 years</strong> old and I am completing this survey with my child

Q41 The survey is divided in **three parts**: PART 1: to be **completed** by the **adult** PART 2: to be **completed** by the **child with** the **help of the adult** PART 3: to be **completed by the child**

End of Block: Block 4 -Survey description for adults

Start of Block: Block 5 - Demographics for adults

Display this question:

If Please read the Information Sheet to learn more about this survey. We have designed a document wi... = I AGREE for myself and my child to take part in this survey

Q40 **PART 1 - To be completed by the adult**

Display this question:

If Please read the Information Sheet to learn more about this survey. We have designed a document wi... = I AGREE for myself and my child to take part in this survey

Q3 How old is your child?

▼ 3 (1) ... 18 (16)

Display this question:

If Please read the Information Sheet to learn more about this survey. We have designed a document wi... = I AGREE for myself and my child to take part in this survey

Q4 What is your child's gender?

- Male (1)
- Female (2)
- Prefer not to say (3)
- Transgender (4)
- Non-binary (5)

Display this question:

If Please read the Information Sheet to learn more about this survey. We have designed a document wi... = I AGREE for myself and my child to take part in this survey

|  |
| --- |

Q1 In which country do you currently reside?

▼ Afghanistan (1) ... Zimbabwe (1357)

End of Block: Block 5 - Demographics for adults

Start of Block: Block 6 - Demographics for young person

Display this question:

If Before starting the survey, please learn more about this survey.  Depending on your age, click on... = I am the adult and I AGREE for my child to take part in this survey

Or Before starting the survey, please learn more about this survey.  Depending on your age, click on... =

Q25 How old are you?

▼ 12 (2) ... 18 (8)

Display this question:

If Before starting the survey, please learn more about this survey.  Depending on your age, click on... = I am the adult and I AGREE for my child to take part in this survey

Or Before starting the survey, please learn more about this survey.  Depending on your age, click on... =

Q26 What is your gender?

- Male (1)
- Female (2)
- Non-binary (4)
- Transgender (5)
- Prefer not to say (3)

Display this question:

If Before starting the survey, please learn more about this survey.  Depending on your age, click on... = I am the adult and I AGREE for my child to take part in this survey

Or Before starting the survey, please learn more about this survey.  Depending on your age, click on... =

|  |
| --- |

Q1 In which country do you live?

▼ Afghanistan (1) ... Zimbabwe (1357)

End of Block: Block 6 - Demographics for young person

Start of Block: Block 7 - Medicines taken for adults

Display this question:

If Please read the Information Sheet to learn more about this survey. We have designed a document wi... = I AGREE for myself and my child to take part in this survey

Q7 Does your child have any problem with colour vision (e.g. colour-blindness)?

- Yes (1)
- No (2)
- I don't know (3)

Display this question:

If Please read the Information Sheet to learn more about this survey. We have designed a document wi... = I AGREE for myself and my child to take part in this survey

Q8 Does your child have a chronic illness? This is a long-term illness  diagnosed by a doctor that lasts three months or more.

- Yes, my child has a long-term illness (1)
- No, my child doesn't have a long-term illness (2)
- I don't know (3)

Display this question:

If Please read the Information Sheet to learn more about this survey. We have designed a document wi... = I AGREE for myself and my child to take part in this survey

And Does your child have a chronic illness? This is a long-term illness diagnosed by a doctor that la... != Yes, my child has a long-term illness

Q9 Has your child ever taken **medicines by mouth (orally)**?

- Yes (1)
- No (2)
- I don't know (3)

Display this question:

If Please read the Information Sheet to learn more about this survey. We have designed a document wi... = I AGREE for myself and my child to take part in this survey

And Does your child have a chronic illness? This is a long-term illness diagnosed by a doctor that la... = Yes, my child has a long-term illness

Q60 Does your child currently take any **medicine by mouth (orally)**? OR has your child ever taken medicines **by mouth**?

- Yes (1)
- No (2)
- I don't know (3)

End of Block: Block 7 - Medicines taken for adults

Start of Block: Block 8 - Medicines taken for young person

Display this question:

If Before starting the survey, please learn more about this survey.  Depending on your age, click on... =

Or Before starting the survey, please learn more about this survey.  Depending on your age, click on... = I am the adult and I AGREE for my child to take part in this survey

Q28 Do you have any problem with colour vision? This means that you see colours differently than most people.

- Yes (1)
- No (2)
- I don't know (3)

Display this question:

If Before starting the survey, please learn more about this survey.  Depending on your age, click on... =

Or Before starting the survey, please learn more about this survey.  Depending on your age, click on... = I am the adult and I AGREE for my child to take part in this survey

Q29 Do you have a chronic illness? This is a long-term illness diagnosed by a doctor that lasts 3 months or more.

- Yes, I have a illness that lasts 3 months or more (1)
- No, I don't have a long-term illness (2)
- I don't know (3)

Display this question:

If Before starting the survey, please learn more about this survey.  Depending on your age, click on... =

Or Before starting the survey, please learn more about this survey.  Depending on your age, click on... = I am the adult and I AGREE for my child to take part in this survey

And Do you have a chronic illness? This is a long-term illness diagnosed by a doctor that lasts 3 mon... = Yes, I have a illness that lasts 3 months or more

Q30 Do you currently take any medicine **by mouth**? OR have you ever taken medicines **by mouth**?

- Yes (1)
- No (2)
- I don't know (3)

Display this question:

If Before starting the survey, please learn more about this survey.  Depending on your age, click on... =

Or Before starting the survey, please learn more about this survey.  Depending on your age, click on... = I am the adult and I AGREE for my child to take part in this survey

And Do you have a chronic illness? This is a long-term illness diagnosed by a doctor that lasts 3 mon... != Yes, I have a illness that lasts 3 months or more

Q61 Have you ever taken medicines **by mouth**?

- Yes (1)
- No (2)
- I don't know (3)

End of Block: Block 8 - Medicines taken for young person

Start of Block: Block 9 - Number of medicines for adults

Display this question:

If Please read the Information Sheet to learn more about this survey. We have designed a document wi... = I AGREE for myself and my child to take part in this survey

And Does your child currently take any medicine by mouth (orally)? OR has your child ever taken medic... = Yes

Q54 Specify the **number of medicines** that your child takes (OR took) **by mouth daily** (e.g. 1, 2, 3 etc.)

________________________________________________________________

End of Block: Block 9 - Number of medicines for adults

Start of Block: Block 10 - Oral medicines chronic illness for adults

Display this question:

If Please read the Information Sheet to learn more about this survey. We have designed a document wi... = I AGREE for myself and my child to take part in this survey

And Does your child currently take any medicine by mouth (orally)? OR has your child ever taken medic... = Yes

Q10 The following questions refer to the medicine(s) that your child takes (OR took) by mouth. **If your child takes (OR took) MORE than ONE medicine by mouth, we will ask you the same questions for each medicine taken.** **${lm://Field/1}${lm://Field/2} medicine taken by mouth** Do you know if this is: a **prescription medicine** (*a medicine that was prescribed by your doctor*) OR an **over-the-counter medicine** (*a medicine that can be bought without a prescription at the pharmacy or supermarket, e.g. vitamins*)?

- Prescription medicine (1)
- Over-the-counter medicine (2)
- I don't know (3)

Display this question:

If Please read the Information Sheet to learn more about this survey. We have designed a document wi... = I AGREE for myself and my child to take part in this survey

And Does your child currently take any medicine by mouth (orally)? OR has your child ever taken medic... = Yes

Q11 Select the **type** of medicine your child takes (OR took) from the list below.

- Capsule (2)
- Tablet (1)
- Orodispersible tablet - a tablet that melts in the mouth (4)
- Minitablets (7)
- Granules / powders - these are usually dispersed in water or other liquid or mixed with food (6)
- Effervescent tablet - a type of tablet that dissolves in water to create fizzy or bubbling solution (5)
- Liquid medicine (3)
- Orodispersible film - a film that melts in the mouth (8)

| Page Break |  |
| --- | --- |

Display this question:

If Please read the Information Sheet to learn more about this survey. We have designed a document wi... = I AGREE for myself and my child to take part in this survey

And Does your child currently take any medicine by mouth (orally)? OR has your child ever taken medic... = Yes

Q43 **PART 2 -  From now on please ask your child for the answer**

Display this question:

If Please read the Information Sheet to learn more about this survey. We have designed a document wi... = I AGREE for myself and my child to take part in this survey

And Does your child currently take any medicine by mouth (orally)? OR has your child ever taken medic... = Yes

Q13 What is (or was) the **colour** of this medicine? Please ask them directly if possible

________________________________________________________________

Display this question:

If Please read the Information Sheet to learn more about this survey. We have designed a document wi... = I AGREE for myself and my child to take part in this survey

And Does your child currently take any medicine by mouth (orally)? OR has your child ever taken medic... = Yes

Q14 Does your child **like its colour**? Please ask them directly, if possible

|  | (4) | (5) | (6) | (7) | (8) |
| --- | --- | --- | --- | --- | --- |
| Let them let them pick a face! (6) |  |  |  |  |  |

Display this question:

If Please read the Information Sheet to learn more about this survey. We have designed a document wi... = I AGREE for myself and my child to take part in this survey

And Does your child currently take any medicine by mouth (orally)? OR has your child ever taken medic... = Yes

Q15 Does (did) your child like the **taste** of this medicine?  Please ask them directly if possible

|  | (1) | (2) | (3) | (4) | (5) |
| --- | --- | --- | --- | --- | --- |
| Let them pick a face! (1) |  |  |  |  |  |

Display this question:

If Please read the Information Sheet to learn more about this survey. We have designed a document wi... = I AGREE for myself and my child to take part in this survey

And Does your child currently take any medicine by mouth (orally)? OR has your child ever taken medic... = Yes

Q16 **Overall** does (did) your child like this medicine?  Please ask them directly if possible

|  | (1) | (2) | (3) | (4) | (5) |
| --- | --- | --- | --- | --- | --- |
| Let them pick a face! (1) |  |  |  |  |  |

End of Block: Block 10 - Oral medicines chronic illness for adults

Start of Block: Block 11 - Oral medicines no illness for adults

Display this question:

If Please read the Information Sheet to learn more about this survey. We have designed a document wi... = I AGREE for myself and my child to take part in this survey

And Has your child ever taken medicines by mouth (orally)? = Yes

Q1 The following questions refer to the last medicine your child took by mouth. Do you remember if this was: a **prescription medicine** (*a medicine that was prescribed by your doctor*) OR an **over-the-counter medicine** (*a medicine that can be bought without a prescription at the pharmacy or supermarket, e.g. vitamins*)?

- Prescription medicine (1)
- Over-the-counter medicine (2)
- I don't know (3)

Display this question:

If Please read the Information Sheet to learn more about this survey. We have designed a document wi... = I AGREE for myself and my child to take part in this survey

And Has your child ever taken medicines by mouth (orally)? = Yes

Q2 Select the **type** of medicine your child took from the list below.

- Capsule (2)
- Tablet (1)
- Orodispersible tablet - a tablet that melts in the mouth (4)
- Minitablets (7)
- Granules / powders - these are usually dispersed in water or other liquid or mixed with food (6)
- Effervescent tablet - a type of tablet that dissolves in water to create a fizzy or bubbling solution (5)
- Liquid medicine (3)
- Orodispersible film - a film that melts in the mouth (8)

| Page Break |  |
| --- | --- |

Display this question:

If Please read the Information Sheet to learn more about this survey. We have designed a document wi... = I AGREE for myself and my child to take part in this survey

And Has your child ever taken medicines by mouth (orally)? = Yes

Q3 **PART 2 -  From now on please ask your child for the answer**

Display this question:

If Please read the Information Sheet to learn more about this survey. We have designed a document wi... = I AGREE for myself and my child to take part in this survey

And Has your child ever taken medicines by mouth (orally)? = Yes

Q4 What was the **colour** of the medicine your child took? Please ask them directly if possible

________________________________________________________________

Display this question:

If Please read the Information Sheet to learn more about this survey. We have designed a document wi... = I AGREE for myself and my child to take part in this survey

And Has your child ever taken medicines by mouth (orally)? = Yes

Q5 Did your child **like its colour**? Please ask them directly, if possible

|  | (4) | (5) | (6) | (7) | (8) |
| --- | --- | --- | --- | --- | --- |
| Let them let them pick a face! (6) |  |  |  |  |  |

Display this question:

If Please read the Information Sheet to learn more about this survey. We have designed a document wi... = I AGREE for myself and my child to take part in this survey

And Has your child ever taken medicines by mouth (orally)? = Yes

Q6 Did your child like the **taste** of this medicine?  Please ask them directly if possible

|  | (1) | (2) | (3) | (4) | (5) |
| --- | --- | --- | --- | --- | --- |
| Let them pick a face! (1) |  |  |  |  |  |

Display this question:

If Please read the Information Sheet to learn more about this survey. We have designed a document wi... = I AGREE for myself and my child to take part in this survey

And Has your child ever taken medicines by mouth (orally)? = Yes

Q7 **Overall** did your child like this medicine?  Please ask them directly if possible

|  | (1) | (2) | (3) | (4) | (5) |
| --- | --- | --- | --- | --- | --- |
| Let them pick a face! (1) |  |  |  |  |  |

End of Block: Block 11 - Oral medicines no illness for adults

Start of Block: Block 12 - Number of medicines for young person

Display this question:

If Before starting the survey, please learn more about this survey.  Depending on your age, click on... =

Or Before starting the survey, please learn more about this survey.  Depending on your age, click on... = I am the adult and I AGREE for my child to take part in this survey

And Do you currently take any medicine by mouth? OR have you ever taken medicines by mouth? = Yes

Q55 **How many** medicines do you take (OR took)**by mouth daily**? (e.g. 1, 2, 3, etc)

________________________________________________________________

End of Block: Block 12 - Number of medicines for young person

Start of Block: Block 13 - Oral medicines chronic illness for young person

Display this question:

If Before starting the survey, please learn more about this survey.  Depending on your age, click on... =

Or Before starting the survey, please learn more about this survey.  Depending on your age, click on... = I am the adult and I AGREE for my child to take part in this survey

And Do you currently take any medicine by mouth? OR have you ever taken medicines by mouth? = Yes

Q1 The next questions are about the **medicine that you are taking (OR took) by mouth.** **If you take (or took) MORE than ONE medicine by mouth, you will be asked the same questions for each medicine**. **${lm://Field/1}${lm://Field/2} medicine** Do you know if this is: a **prescription medicine** ( a medicine that was prescribed by your doctor) OR an **over-the-counter medicine** (a medicine that can be bought without a prescription at the pharmacy or supermarket, e.g. vitamins)?

- Prescription medicine (1)
- Over-the-counter medicine (2)
- I don't know (3)

Display this question:

If Before starting the survey, please learn more about this survey.  Depending on your age, click on... =

Or Before starting the survey, please learn more about this survey.  Depending on your age, click on... = I am the adult and I AGREE for my child to take part in this survey

And Do you currently take any medicine by mouth? OR have you ever taken medicines by mouth? = Yes

Q2 Select from the list below the type of medicine **you are taking (OR took)**.

- Capsule (2)
- Tablet (1)
- Orodispersible tablet (tablet that melts in the mouth) (4)
- Minitablets (7)
- Granules / powders - these are usually dispersed in water or other liquid or mixed with food (6)
- Effervescent tablet - a type of tablet that dissolves in water to create a fizzy or bubbling solution (5)
- Liquid medicine (3)
- Orodispersible film (film that melts in the mouth) (8)

Display this question:

If Before starting the survey, please learn more about this survey.  Depending on your age, click on... =

Or Before starting the survey, please learn more about this survey.  Depending on your age, click on... = I am the adult and I AGREE for my child to take part in this survey

And Do you currently take any medicine by mouth? OR have you ever taken medicines by mouth? = Yes

Q3 What is (or was) the **colour** of this medicine?

________________________________________________________________

Display this question:

If Before starting the survey, please learn more about this survey.  Depending on your age, click on... =

Or Before starting the survey, please learn more about this survey.  Depending on your age, click on... = I am the adult and I AGREE for my child to take part in this survey

And Do you currently take any medicine by mouth? OR have you ever taken medicines by mouth? = Yes

Q4 Do (or did) you **like its colour**?

|  | (1) | (2) | (3) | (4) | (5) |
| --- | --- | --- | --- | --- | --- |
| Pick a face! (1) |  |  |  |  |  |

Display this question:

If Before starting the survey, please learn more about this survey.  Depending on your age, click on... = I am the adult and I AGREE for my child to take part in this survey

Or Before starting the survey, please learn more about this survey.  Depending on your age, click on... =

And Do you currently take any medicine by mouth? OR have you ever taken medicines by mouth? = Yes

Q5 Do (or did) you like the **taste** of this medicine?

|  | (1) | (2) | (3) | (4) | (5) |
| --- | --- | --- | --- | --- | --- |
| Pick a face! (1) |  |  |  |  |  |

Display this question:

If Before starting the survey, please learn more about this survey.  Depending on your age, click on... =

Or Before starting the survey, please learn more about this survey.  Depending on your age, click on... = I am the adult and I AGREE for my child to take part in this survey

And Do you currently take any medicine by mouth? OR have you ever taken medicines by mouth? = Yes

Q6 **Overall** do (or did) you like this medicine?

|  | (1) | (2) | (3) | (4) | (5) |
| --- | --- | --- | --- | --- | --- |
| Pick a face! (1) |  |  |  |  |  |

End of Block: Block 13 - Oral medicines chronic illness for young person

Start of Block: Block 14 - Oral medicines no illness for young person

Display this question:

If Before starting the survey, please learn more about this survey.  Depending on your age, click on... =

Or Before starting the survey, please learn more about this survey.  Depending on your age, click on... = I am the adult and I AGREE for my child to take part in this survey

And Have you ever taken medicines by mouth?  = Yes

Q1 The next questions are about the **last medicine** that you took **by mouth.** Do you remember if this is was: a **prescription medicine** ( *a medicine that was prescribed by your doctor*) OR an **over-the-counter medicine** (*a medicine that can be bought without a prescription at the pharmacy or supermarket, e.g. vitamins*)?

- Prescription medicine (1)
- Over-the-counter medicine (2)
- I don't know (3)

Display this question:

If Before starting the survey, please learn more about this survey.  Depending on your age, click on... =

Or Before starting the survey, please learn more about this survey.  Depending on your age, click on... = I am the adult and I AGREE for my child to take part in this survey

And Have you ever taken medicines by mouth?  = Yes

Q2 Do you remember the type of medicine **you took?** Select from the list below.

- Capsule (2)
- Tablet (1)
- Orodispersible tablet - a tablet that melts in the mouth (4)
- Minitablets (7)
- Granules / powders - these are usually dispersed in water or other liquid or mixed with food (6)
- Effervescent tablet - a type of tablet that dissolves in water to create a fizzy or bubbling solution (5)
- Liquid medicine (3)
- Orodispersible film - a film that melts in the mouth (8)

Display this question:

If Before starting the survey, please learn more about this survey.  Depending on your age, click on... =

Or Before starting the survey, please learn more about this survey.  Depending on your age, click on... = I am the adult and I AGREE for my child to take part in this survey

And Have you ever taken medicines by mouth?  = Yes

Q3 What was the **colour** of this medicine?

________________________________________________________________

Display this question:

If Before starting the survey, please learn more about this survey.  Depending on your age, click on... =

Or Before starting the survey, please learn more about this survey.  Depending on your age, click on... = I am the adult and I AGREE for my child to take part in this survey

And Have you ever taken medicines by mouth?  = Yes

Q4 Do you **like its colour**?

|  | (1) | (2) | (3) | (4) | (5) |
| --- | --- | --- | --- | --- | --- |
| Pick a face! (1) |  |  |  |  |  |

Display this question:

If Before starting the survey, please learn more about this survey.  Depending on your age, click on... =

Or Before starting the survey, please learn more about this survey.  Depending on your age, click on... = I am the adult and I AGREE for my child to take part in this survey

And Have you ever taken medicines by mouth?  = Yes

Q5 Did you like the **taste** of this medicine?

|  | (1) | (2) | (3) | (4) | (5) |
| --- | --- | --- | --- | --- | --- |
| Pick a face! (1) |  |  |  |  |  |

Display this question:

If Before starting the survey, please learn more about this survey.  Depending on your age, click on... =

Or Before starting the survey, please learn more about this survey.  Depending on your age, click on... = I am the adult and I AGREE for my child to take part in this survey

And Have you ever taken medicines by mouth?  = Yes

Q6 **Overall** did you like this medicine?

|  | (1) | (2) | (3) | (4) | (5) |
| --- | --- | --- | --- | --- | --- |
| Pick a face! (1) |  |  |  |  |  |

End of Block: Block 14 - Oral medicines no illness for young person

Start of Block: Block 15 - Questions for children

Display this question:

If Please read the Information Sheet to learn more about this survey. We have designed a document wi... = I AGREE for myself and my child to take part in this survey

And Who is completing this questionnaire? != I am <strong>12 to 18</strong> years old

Q31 **PART 3 - please let your child complete this part on its own (if possible)** If your child is unable to complete the survey on their own and they require your assistance, please tick the box below.

- I am completing this part on behalf of my child (4)

Q59 Would you like taking medicines that don't have any colour, e.g. white or transparent medicines like those in these photos?

- Yes (2)
- No (4)
- I don't know (3)

| Page Break |  |
| --- | --- |

Q32 If you could **pick a colour** for this **liquid medicine**, which colour would you like? Choose one from the colours below!

- Blue (1)
- Brown (2)
- Clear / transparent (3)
- Green (4)
- Orange (5)
- Pink (6)
- Red (7)
- White (8)
- Yellow (9)
- Purple (10)

Q33 Can you tell me **why** you chose this colour?  *I choose this colour because ...*

________________________________________________________________

Q34 What **taste** do you think this colour would be?

________________________________________________________________

| Page Break |  |
| --- | --- |

Q35 If you could **pick a colour** for this**tablet**, which colour would you like? Choose one from the colours below!

- Yellow (1)
- White (2)
- Purple (3)
- Orange (4)
- Green (5)
- Brown (6)
- Blue (8)
- Red (9)
- Pink (10)

Q36 Can you tell me **why** you chose this colour?  *I choose this colour because ...*

________________________________________________________________

Q37 What **taste** do you think this colour would be?

________________________________________________________________

# Supplementary Figures and Tables

Supplementary Table 1. Top three colours selected for hypothetical liquid and solid medicines by age-groups.

|  | Hypothetical liquid medicine | | Hypothetical solid medicine | |
| --- | --- | --- | --- | --- |
| Age-group | **Colour** | **%** | **Colour** | **%** |
| 3 to 6 years | Pink | 21.7 | Pink | 19.4 |
|  | Purple | 17.4 | Purple / red | 17.9 |
|  | Red | 14.5 | Blue / white | 10.4 |
| 7 to 11 years | Blue | 24.4 | White | 18.2 |
|  | Pink | 17.9 | Blue | 16.9 |
|  | Purple / orange | 12.8 | Purple / red | 13.0 |
| 12 to 18 years | Pink | 25.2 | White | 38.7 |
|  | Colourless | 22.5 | Pink | 19.8 |
|  | Blue | 12.6 | Blue | 11.5 |


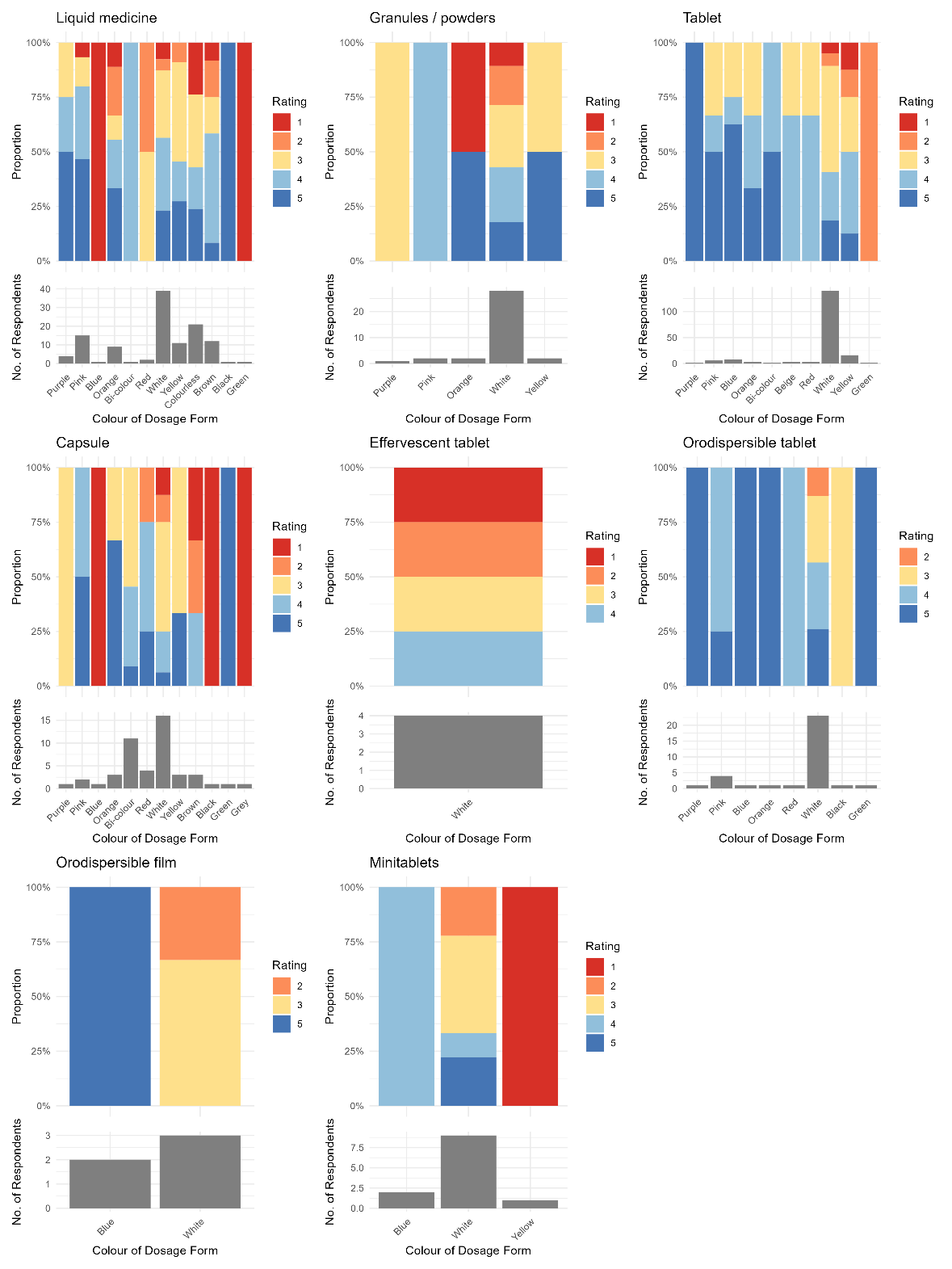


**Supplementary Figure 1.** Colour ratings for the most recently taken oral medicine by participants, along with the number of respondents who took a specific dosage form with a particular colour.
